# Combination of exercise training and erythropoietin prevents cancer-induced muscle alterations

**DOI:** 10.18632/oncotarget.6439

**Published:** 2015-11-30

**Authors:** Fabrizio Pin, Silvia Busquets, Miriam Toledo, Andrea Camperi, Francisco J. Lopez-Soriano, Paola Costelli, Josep M. Argilés, Fabio Penna

**Affiliations:** ^1^ Department of Clinical and Biological Sciences, University of Torino, Torino, Italy; ^2^ Cancer Research Group, Departament de Bioquímica i Biologia Molecular, Facultat de Biologia, Universitat de Barcelona, Barcelona, Spain; ^3^ Institut de Biomedicina de la Universitat de Barcelona (IBUB), Barcelona, Spain

**Keywords:** cancer cachexia, exercise training, erythropoietin, PGC-1α, mitochondria, Pathology Section

## Abstract

Cancer cachexia is a syndrome characterized by loss of skeletal muscle mass, inflammation, anorexia and anemia, contributing to patient fatigue and reduced quality of life. In addition to nutritional approaches, exercise training (EX) has been proposed as a suitable tool to manage cachexia. In the present work the effect of mild exercise training, coupled to erythropoietin (EPO) administration to prevent anemia, has been tested in tumor-bearing mice. In the C26 hosts, acute exercise does not prevent and even worsens muscle wasting. Such pattern is prevented by EPO co-administration or by the adoption of a chronic exercise protocol. EX and EPO co-treatment spares oxidative myofibers from atrophy and counteracts the oxidative to glycolytic shift, inducing PGC-1α. LLC hosts are responsive to exercise and their treatment with the EX-EPO combination prevents the loss of muscle strength and the onset of mitochondrial ultrastructural alterations, while increases muscle oxidative capacity and intracellular ATP content, likely depending on PGC-1α induction and mitophagy promotion. Consistently, muscle-specific PGC-1α overexpression prevents LLC-induced muscle atrophy and Atrogin-1 hyperexpression. Overall, the present data suggest that low intensisty exercise can be an effective tool to be included in combined therapeutic approaches against cancer cachexia, provided that anemia is coincidently treated in order to enhance the beneficial action of exercise.

## INTRODUCTION

Cancer cachexia is a multifactorial syndrome frequently occurring in association with different types of cancers. It is characterized by a complex interplay of factors, resulting in poor quality of life, decreased tolerance to anticancer therapies and reduced survival [1]. The pathophysiology of cachexia includes increased protein catabolism, systemic inflammation, hormonal disturbances and down-regulation of anabolic signals. Depletion of the skeletal muscle mass, not necessarily associated with the loss of fat mass, is the most relevant feature of cachexia. Such a pattern results in progressive reduction of muscle strength, endurance and exercise capacity [2], and cannot be fully reversed by conventional nutritional support.

A frequent comorbidity of cancer is anemia (hemoglobin level < 12 g/dl), that occurs in approximately 40% of cancer patients. Its incidence depends on a number of variables such as tumor site and origin, progression, stage, extent of disease and cancer treatment [3]. Anemia potentially promotes the progression of wasting in cachectic patients [4]. In this regard, a direct correlation between hemoglobin levels and the quality of life of cancer patients has been established, the onset of anemia being related to decreased functional status and survival [3]. Anemia and muscle wasting are mainly responsible for fatigue, hampering patient daily activities and independent life. In peripheral tissues, skeletal muscle included, anemia can cause hypoxia, with consequent intracellular acidification and reduction of oxidative metabolism. These changes might result, respectively, in increased protein degradation and cell damage, eventually leading to muscle wasting [5].

The most effective pharmacological treatment for anemia is erythropoietin (EPO), an endogenous cytokine/hormone able to stimulate erythropoiesis. EPO acts binding to its specific receptor (EPO-R), belonging to the family of cytokine receptors characterized by a single transmembrane domain [6]. EPO has pleiotropic functions, since EPO-R expression is not restricted to hematopoietic cells, but is present in heart [7], skeletal muscle [6] and adipose tissue [8]. In this regard, in the skeletal muscle of EPO-deficient mice the expression of genes related to mitochondrial function is low, while genes involved in proteolysis and hypoxia are overexpressed, suggesting that EPO plays a relevant role in muscle tissue [9]. Consistently, EPO administration to rats rapidly stimulates glucose metabolism and muscle anabolism [10]. Recently, EPO and EPO-R have been involved in muscle regeneration. Indeed, mice with high circulating EPO show an improved recovery from muscle injury and, conversely, muscles lacking EPO-R display an increased susceptibility to cardiotoxin-induced damage [11]. Finally, several studies demonstrate that EPO promotes a shift from glycolytic to oxidative metabolism [6]. A protective action of EPO against muscle wasting could be inferred from these observations, glycolytic myofibers being those preferentially affected in cancer cachexia [12].

The mechanisms underlying cachexia are still poorly understood, and the availability of effective interventions is quite limited. In this regard, exercise training has been proposed as a mean to improve the quality of life of cachectic patients [13]. Indeed, the beneficial effect of exercise in counteracting fatigue and exhaustion has been extensively demonstrated [14]. According to the adopted protocol, exercise training can either increased muscle mass or improve cardiovascular function, stimulating specific pathways; as a consequence, muscle atrophy or fatigue in cancer patients could be improved [13, 15]. Since tumor growth induces several metabolic changes in the skeletal muscle such as reduced ATP synthesis, mitochondrial alterations and shift from oxidative to glycolytic metabolism [4], endurance exercise might well be proposed to reverse or attenuate all these alterations, resulting in prevention/delay of cancer-induced muscle wasting.

Aim of the present study was to verify whether the adoption of a low intensity endurance exercise protocol could counteract the loss of muscle mass and function occurring in experimental cancer cachexia. Exercise has been associated with EPO administration in order to test if, beyond affecting anemia, EPO could further improve exercise effects on muscle wasting.

## RESULTS

We previously showed that moderate aerobic exercise associated with eicosapentaenoic acid administration partially prevented the loss of muscle mass and strength in mice bearing the Lewis Lung Carcinoma (LLC; [16]), while the same exercise protocol worsened muscle wasting in mice hosting the C26 carcinoma [17]. Anemia occurs in both LLC or C26 hosts [8] and could negatively modulate the response to exercise. To clarify this point, in the present study two different exercise protocols have been associated with EPO administration to tumor-bearing mice.

**Figure 1:**
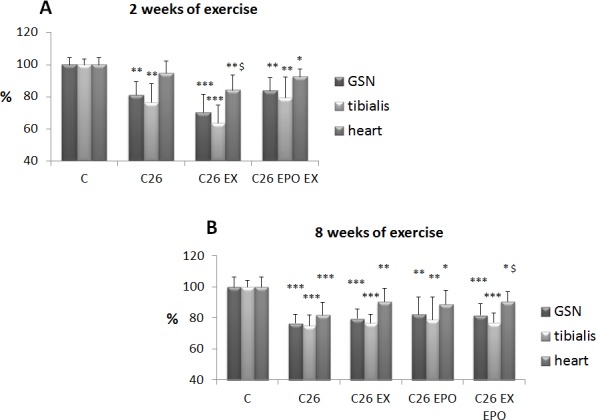
Exercise training and EPO do not prevent C26-induced muscle loss **A.** Gastrocnemius (GSN), tibialis and heart weight in control (C), and C26-bearing mice (C26). C26 groups were subdivided in sedentary, exercised (EX), and exercised EPO-treated (EX EPO). **B.** GSN, tibialis and heart weight in control (C), and C26-bearing mice (C26). C26 groups were subdivided in untreated, exercised (EX), EPO-treated (EPO) and exercised EPO-treated (EX EPO) for eight weeks (of exercise). Data (mean±SD) are expressed as percentages of C. Significance of the differences: *p < 0,05 *vs* C, **p < 0,01 *vs* C, ***p < 0,001 *vs* C, $*p* < 0,05 *vs* C26.

Confirming previous observations [18], the growth of the C26 tumor results in reduced body weight, food intake, muscle strength and mass (Figure 1A, 1B, S1, S2). Such a wasting pattern cannot be rescued, or can be even worsened, by two weeks of low intensity endurance exercise, while the association with EPO improves body and heart weight in comparison to trained C26 hosts (Figure 1A, S1A). In a second experiment the animals have been exercised for eight weeks (6 before and 2 after tumor injection). Such a schedule likely results in mice adaptation to exercise, allowing to test the susceptibility to cachexia in mildly trained animals. At sacrifice, 14 days after tumor implantation, no differences in body and muscle weight can be observed in tumor-bearing mice, irrespective of the treatment (exercise with or without EPO; Figure 1B, Figure S2A), while the association of exercise with EPO partially prevents C26-induced heart depletion (Figure 1B). While the two week exercise protocol does not affect, or even reduces, food intake in the C26 hosts (Figure S1B), eight week training prevents C26-induced anorexia (Figure S2B). As for muscle function, the reduced muscle strength found in tumor-bearing animals is not modified by the two week exercise protocol, independently of EPO treatment (Figure S1C). By contrast, the eight week training improves grip strength, only when combined with EPO (Figure S2C). In both experiments EPO administration restores hematocrit to normal values (Figure S3B, S4B).

C26-bearing animals present with spleen hyperplasia (Figure S3A, S4A) and high circulating IL-6 levels (Figure S3C, S4C). While spleen mass is reduced by both exercise protocols (two or eight weeks; Figure S3A, S4A), only acute (2 weeks) exercise decreases IL-6 concentrations, an effect that is enhanced by co-treatment with EPO (Figure S3C, S4C). As for the other tissues examined, the two week exercise protocol does not affect liver mass, even when associated with EPO administration, while the combination EPO-exercise partially prevents the loss of adipose tissue (Figure S3A), confirming previous results [8]. The eight week training produces a comparable, though not totally overlapping, pattern (Figure S4A).

**Figure 2:**
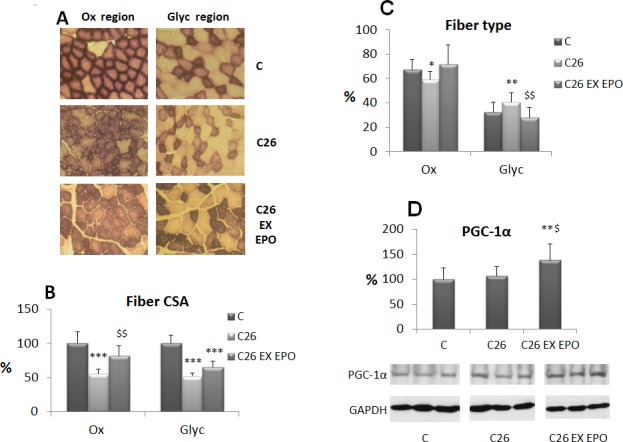
Exercise training and EPO counteract oxidative fiber atrophy and glycolytic shift stimulating PGC-1α expression **A.** SDH (succinate dehydrogenase) staining in cross sections of tibialis muscle from control (C), C26-bearing (C26) and C26 exercised EPO-treated (EX EPO) mice (2 weeks of exercise). The two micrographs for each group represent two regions with distinct frequency of oxidative (Ox) and glycolytic (Glyc) fibers. **B.** Morphometric analysis of myofiber CSA (cross-sectional area) performed on SDH stained sections. Data (mean±SD) are expressed as percentages of controls. **C.** Quantification of fiber type frequency in the tibialis muscle. Data (mean±SD) are expressed as relative percentages. **D.** PGC-1α nuclear protein expression in the tibialis muscles. Densitometric quantifications were normalized according to GAPDH levels. Data (mean±SD) are expressed as percentages of controls. Significance of the differences: *p < 0,05 *vs* C, **p < 0,01 *vs* C, ***p < 0,001 *vs* C, $*p* < 0,05 *vs* C26, $$*p* < 0,01 *vs* C26.

In order to check if two weeks of low intensity endurance exercise and EPO treatment could affect muscle fiber type composition and size, myofiber CSA has been assessed on SDH stained section (Figure 2A), virtually separating the tibialis muscle into two zones, according to the prevalence of oxidative or glycolytic fibers. C26 growth leads to a significant CSA reduction in both oxidative and glycolytic fibers (Figure 2B, S5), while the combination of exercise and EPO restores CSA in the former, though not in the latter. The presence of the tumor also drives an oxidative to glycolytic fiber shift, that is reversed by the combined treatment (Figure 2C). Finally, the expression levels of PGC-1α, a factor involved in mitochondrial biogenesis and function, have been evaluated in the skeletal muscle of treated and untreated tumor-bearing mice. The results show that PGC-1α expression does not change between controls and C26 hosts, despite previous observations showing a reduction in the latter [18]. Consistently with the increased number of oxidative myofibers, the combined treatment EX-EPO significantly enhances PGC-1α expression in the skeletal muscle of the C26-bearing mice (Figure 2D).

**Figure 3:**
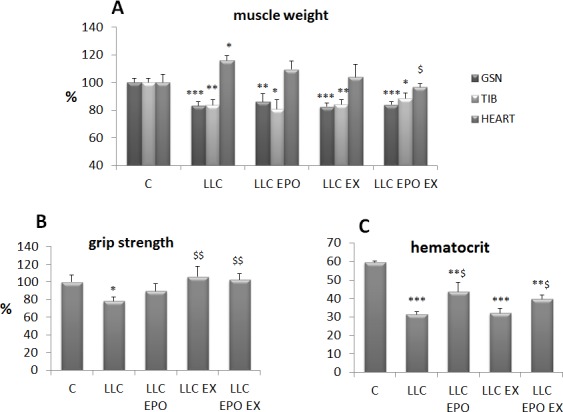
Exercise training and EPO partially prevent cachexia in LLC-bearing mice Gastrocnemius (*GSN*), tibialis and heart weight **A.**, voluntary grasping strength **B.** and hematocrit **C.**, in control (C) and LLC-bearing mice (LLC). LLC groups were subdivided in sedentary, EPO-treated (EPO), exercised (EX) and exercised EPO-treated (EX EPO)for four weeks. Data (mean±SD) expressed as percentages of controls except for hematocrit (absolute values). Significance of the differences: *p < 0,05 *vs* C, **p < 0,01 *vs* C, ***p < 0,001 *vs* C, $*p* < 0,05 *vs* LLC, $$*p* < 0,01 *vs* LLC.

Since exercise and EPO partially improve cachexia in the C26 hosts, such combined approach has been tested also in the LLC-bearing mice, where anemia is markedly severe. Moreover, four weeks of LLC growth are required to lose animal muscle mass and strength (Figure 3A, 3B), allowing to adopt an exercise protocol more extended than the one used during C26 growth. The results show that the low intensity endurance exercise adopted does not improve muscle mass in LLC-bearing mice (Figure 3A), while it is able to rescue muscle strength (Figure 3B). Cardiac hypertrophy and anemia occur in the LLC hosts (Figure 3A, 3C). The former tends to decrease after exercise or EPO alone and is completely reversed by the combined treatment (Figure 3A), while EPO partially corrects the latter (Figure 3C).

**Figure 4:**
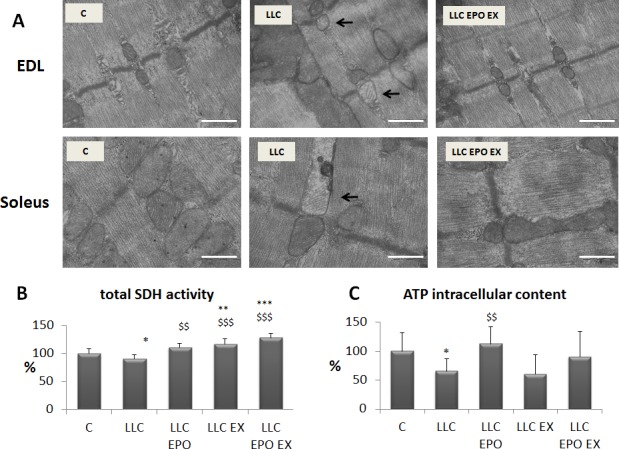
Exercise training and EPO restore mitochondrial morphology in LLC-bearing mice **A.** Transmission electron microscope images of EDL and soleus longitudinal sections, in control (C), LLC-bearing mice (LLC) and exercised EPO-treated (EX EPO). Black arrows indicate altered mitochondria. Scale bar = 500 nm. Quantification of SDH (succinate dehydrogenase) activity in tibilais **B.** and ATP intracellular content in gastrocnemius (GSN) muscle (**C.**, expressed as % of control) of control (C) and LLC-bearing mice (LLC). LLC groups were subdivided in sedentary, EPO-treated (EPO), exercised (EX), and exercised EPO-treated (EX EPO). Significance of the differences: *p < 0,05 *vs* C, **p < 0,01 *vs* C, ***p < 0,001 *vs* C $$*p* < 0,01 *vs* LLC, $$$*p* < 0,001 *vs* LLC.

Muscle mitochondria ultra-structure has been analyzed in the LLC hosts, in order to investigate the effects of exercise and EPO on myofiber energy metabolism. In both EDL (glycolytic) and soleus (oxidative) muscles LLC growth results in the appearance of swollen mitochondria (Figure 4A), similarly to previous observations reported in both C26-bearing mice [19] and AH-130-bearing rats [20]. Other alterations, previously reported in the AH-130 model [20], such as disrupted triads and increased mitochondrial area were observed, however no quantitative data were obtained. Treatment with EPO in association with exercise allows a qualitative (Figure 4A) and, despite the limitations imposed by sample size and area assayed, quantitative (Figure S6) recovery of mitochondrial structure in the skeletal muscle of LLC-bearing mice. In accordance with the above reported mitochondrial damage, myofiber oxidative capacity (SDH activity) is reduced in untreated LLC hosts while is restored to physiological values by both EPO and exercise, alone or combined (Figure 4B). Of interest, exercise, associated or not with EPO, increases SDH activity also in comparison with control mice, suggesting that the tumor-bearing condition is permissive for muscle mitochondrial biogenesis. Dysfunctional mitochondria could lead to impaired intracellular energy stores (ATP), also due to mitochondrial energy uncoupling and increased glycolysis [4]. Consistently, muscle ATP intracellular content in tumor-bearing mice is decreased (Figure 4C). The administration of EPO alone restores the ATP content to control values, while no significant differences can be observed in exercised LLC host, in the absence or in the presence of EPO. This latter observation is quite unexpected, in view of the above reported results showing that EPO associated with exercise promotes mitochondrial structure recovery (see above).

**Figure 5:**
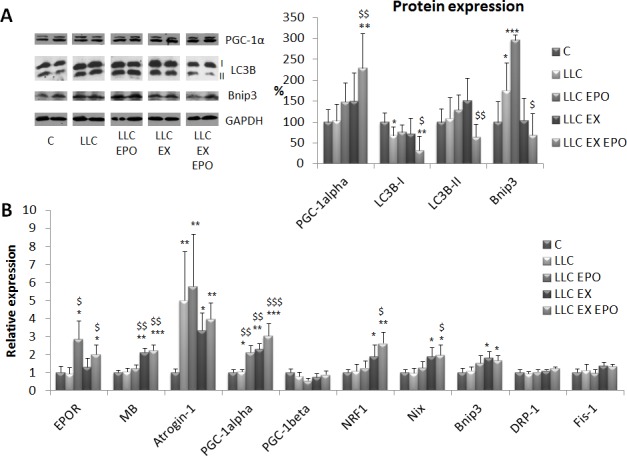
Exercise training and EPO regulate bioenergetics-related gene and protein expression in LLC-bearing mice **A.** Protein expression analysis of PGC-1α, LC3B (either in native or lipidated form, -I and -II, respectively) and Bnip3 corrected for tubulin abundance in gastrocnemius (GSN) muscle of control (C) and LLC-bearing mice (LLC). LLC groups were subdivided in sedentary, EPO-treated (EPO), exercised (EX), and exercised EPO-treated (EX EPO). Data (mean±SD) expressed as % of controls. **B.** Gene expression analysis of EPOR, myoglobin (MB), Atrogin-1, PGC-1alpha, PGC-1beta, NRF1, Nix, Bnip3, DRP-1 and Fis-1 in the tibialis anterior muscle. Specific mRNA abundance was corrected for the mean of TBP (TATA box-binding protein) and β-actin levels on individual samples. Data (mean±SE) are compared by 2-way ANOVA. Significance of the differences: *p < 0,05 *vs* C, **p < 0,01 *vs* C, ***p < 0,001 *vs* C, $*p* < 0,05 *vs* LLC, $$*p* < 0,01 *vs* LLC, $$$*p* < 0,001 *vs* LLC.

From a mechanistic point of view, the recovery of mitochondrial structure and function can be explained again by PGC-1α induction, since both protein (Figure 5A) and mRNA (Figure 5B) levels increase in the muscle of LLC-bearing mice after EX and EPO. Dysfunctional mitochondria are normally degraded by mitophagy [21]. The results shown in the present study demonstrate that in the muscle of the LLC hosts a shift from microtubule-associated (I) to autophagosome-associated LC3B (II) occurs. This is paralleled by increased expression of the mitophagy regulator Bnip3, suggesting the accumulation of damaged mitochondria. In the EX EPO group, both LC3B isoforms are makedly reduced and Bnip3 level is normalized, in line with an efficient mitochondrial clearance (Figure 5A).

The gene expression analysis (Figure 5B) shows that EPO action increases EPO-R, while exercise stimulates myoglobin (MB), probably due to the increased oxygen demand. As a hallmark of protein catabolism, atrogin-1 expression increases in the LLC-bearing mice, and consistently with the unaffected muscle mass, no treatment proves able to prevent such increase. Consistently with PGC-1α levels, exercise, combined or not with EPO, promotes the expression of the mitochondrial biogenesis related gene NRF1, while no changes occur in the levels of PGC-1β mRNA. The promotion of mitochondrial biogenesis by EX and EPO is paralleled by the increase of Nix and Bnip3 mRNA. As for Bnip3, such qualitative difference with protein levels suggests that this regulator of mitophagy can be modulated at the post-translational level. Finally, the transcript levels of two important regulators of mitochondrial fission, namely DRP-1 and Fis-1, that per se could induce muscle atrophy [22], are unchanged in all the experimental conditions.

**Figure 6:**
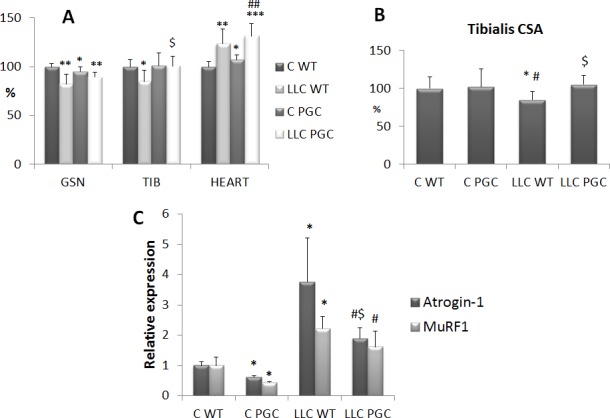
PGC-1α overexpression prevents cachexia in LLC-bearing mice Gastrocnemius (GSN), tibialis (TIB) and heart weight **A.** and tibialis fiber CSA **B.** in female control WT (C WT), PGC (C PGC) and LLC-bearing mice WT (LLC WT) and PGC (LLC PGC). Data (mean±SD) are expressed as percentages of C WT. **C.** Gene expression analysis of Atrogin-1 and MuRF1 transcripts. Specific mRNA abundance was corrected for the mean of TBP (TATA box-binding protein) and β-actin levels on individual samples. Data (means ± SE) are compared by 2-way ANOVA. Significance of the differences: *p< 0,05 *vs* C WT, **p < 0,01 *vs* C WT, ***p < 0,001 *vs* C WT, $*p* < 0,05 *vs* LLC WT, #*p* < 0,05 *vs* C PGC, ##*p* < 0,01 *vs* C PGC.

The effects of endurance exercise can be mimicked by overexpression of PGC-1α, that was shown to prevent atrophy induced by denervation, diabetes, uremia and unloading [23-25]. No information are available about the effectiveness of PGC-1α hyperexpression on cancer-induced muscle wasting. For this reason, male and female transgenic mice overexpressing PGC-1α specifically in the skeletal muscle have been injected with LLC cells. As for male LLC hosts, the resulting tumors are markedly larger than in wild-type littermates (6.08 ± 2.42 g *vs* 3.34 ± 1.52 g, respectively; *n* = 8, *p* = 0.015). By contrast, tumor mass is comparable in wild type or transgenic female mice (3.20 ± 2.30 g *vs* 3.01 ± 2.23 g, respectively; *n* = 8, *p* = 0.874), which is not consistent with previous data [26]. Nonetheless, irrespective of the increased tumor size, the loss of muscle mass is comparable in transgenic and in wild-type male mice (Figure S7A). Not only, myofiber CSA is even larger in transgenic LLC-bearing male mice than in wild-type tumor hosts (Figure S7B). The other way round, PGC-1α overexpression in the skeletal muscle of LLC-bearing female mice restores both tibialis muscle weight and myofiber CSA to the control value (Figure 6A, 6B). A similar, although not complete, rescue can be observed for the GSN mass (Figure 6A), despite this muscle weights markedly less in control PGC-1α than in WT mice, likely due to the distinct fiber composition in transgenic mice presenting almost the totality of oxidative fibers, known to be smaller than the glycolytic ones. As for tumor-induced heart hypertrophy, this was not affected or was even increased by the transgene, PGC-1α mice presenting with a mild heart hypertrophy even in the absence of the tumor (Figure 6A). Consistently with the protection against muscle wasting, in the PGC-1α transgenic females the increase of muscle atrogin-1 levels is less marked than in WT animals (Figure 6C). Of interest, the expression of both muscle-specific ubiquitin ligases atrogin-1 and MuRF1 is reduced in healthy PGC-1α transgenic mice with respect to wild-type controls (Figure 6C).

## DISCUSSION

Effective treatments for the prevention of cachexia are lacking. In this regard, several clinical trials are ongoing, however most of them are based on the use of nutritional and pharmacological (mainly orexigenic) interventions aimed at preventing body weight loss and muscle wasting. By contrast, the effect of exercise training has been explored only marginally [15], even in experimental models of cachexia [17], despite in physiological conditions exercise is the most effective strategy to improve muscle mass and function.

Muscle strength and endurance are markedly impaired in cachexia and many cancer patients experience chronic fatigue, as a consequence of the tumor and/or of the superimposed anti-neoplastic therapies. Chronic fatigue and comorbidities, such as anemia and cardiac dysfunctions, are limiting factors for practicing physical activity [17]. Consistently, the data reported in the present study show that exercise does not improve skeletal muscle wasting in the C26-bearing mice, in which cachexia is associated with anemia (present work) and cardiac alterations [27]. In this regard, cardiovascular perturbations have been reported also in colorectal cancer patients, independently from chemotherapy [28], while anemia has been proposed to depend on a direct action of the tumor, on anti-cancer treatment, or both [3]. Considering that anemia occurs in about 40% of cancer patients or even more after chemotherapy [3] and that potentially limits the beneficial effects exerted by exercise, its correction should be achieved before suggesting exercise as a mean to prevent/correct cachexia. Indeed, our observations show that EPO administration to exercised C26 hosts partially prevents both CSA reduction in oxidative myofibers and the shift from oxidative to glycolytic fiber type.

EPO is not free from side effects, mainly cardiovascular events reported in patients affected by heart or chronic kidney diseases, undergoing prolonged EPO administration [29, 30]. For these reasons, safety evaluation in cachectic cancer patients would be required. However the schedule proposed for EPO administration in the present study is ‘acute’ and would be carried out only in anemic cancer patients to allow exercise practicing.

The mechanisms accounting for EPO effectiveness could go beyond the stimulation of erythropoiesis. Indeed, plenty of data are available about the cardio-protective action of EPO [30], and recent results suggest that different regions of the EPO molecule account for the erythropoietic and the immunomodulatory action [31]. The results shown in the present study demonstrate that both EPO and exercise are required to restore the normal heart weight in LLC-bearing mice, where anemia is more severe than in the C26 hosts, suggesting an additive and coordinated action of the two treatments. However, LLC-bearing mice are hyporesponsive to EPO since the severe anemia could only partially be corrected, possibly due to inflammation that antagonizes erythropoiesis and/or excessive erythrocyte cytolysis[32]. Being the pathogenesis of cachexia a multifactorial process, it is likely that EPO and exercise act on different targets, improving anemia and muscle strength, respectively. In addition, both could positively impinge on heart function.

The inflammatory response is another crucial event in the pathogenesis of cancer cachexia. In this regard, the data shown in the present study confirm previous observations indicating that the levels of circulating IL-6, a pro-inflammatory cytokine that plays a pivotal role in the onset and progression of cachexia [33], markedly increase in tumor-bearing mice. Further supporting the idea that a combined treatment is the best choice to address cancer cachexia, the association between exercise and EPO reduces circulating IL-6, even though such effect is lost when exercise starts long before tumor injection, likely due to an adaptation to the exercise protocol in the absence of a reinforcement in running speed and time. The anti-inflammatory action of exercise is well-established and has been demonstrated in both healthy subjects [34] and cancer patients at early stages of the disease [35]. In cachectic cancer patients, an exercise-mediated anti-inflammatory action would be clinically relevant, since the occurrence of inflammation negatively impinges on diagnosis, staging of cachexia [1, 36], and patient survival [37].

The occurrence of muscle mitochondrial dysfunction in cachexia has been demonstrated by a number of studies [20, 38, 39]. The data here reported confirm that both mitochondrial structure and function are altered in the skeletal muscle of tumor-bearing mice, and that such changes can be rescued by the combined treatment with EPO and exercise. In this regard, several studies suggest that EPO plays a role in the regulation of mitochondrial function: it has been shown to stimulate muscle fat oxidation and to prevent diet-induced obesity in mice [40], to promote muscle mitochondrial biogenesis in rats [41] and to enhance mitochondrial function (oxidative phosphorylation and electron transport capacity) in humans [42]. These observations are consistent with the glycolytic to oxidative myofiber shift observed in the C26-bearing mice as well as with the increased SDH activity occurring in the LLC hosts, both suggesting that the combination of EPO and exercise stimulates mitochondrial biogenesis. Of interest, EPO alone is unable to prevent the accumulation of dysfunctional mitochondria in LLC-bearing mice, suggesting that between EPO and exercise, the latter is leader in driving the action on mitochondria, consistently with a previous report showing reduced mitochondrial damage in exercised doxorubicin-treated rats [43].

In addition to reduced muscle mass, also contractile dysfunction has been proposed to drive both muscle weakness and fatigue occurring in the C26-bearing mice [44]. In this regard, protein hypercatabolism mainly accounts for muscle depletion, while impaired muscle ‘quality’ leads to contractile dysfunction; this latter, in particular, mainly depends on the lack of energy, i.e. ATP intracellular content. So far, most of the interventions proposed to prevent cachexia are aimed to preserve muscle mass, while the rescue of muscle quality, even if seldom pursued, would prevent the loss of ATP production, restoring muscle energetics. No alterations in the efficiency of ATP synthesis have been found in the skeletal muscle of cachectic rats with peritoneal carcinosis [38], while *in vivo* ATP synthesis rate is markedly reduced in LLC-bearing mice [45]. Our results confirm the strong reduction of ATP intracellular storage, that is reversed by EPO administration, though not by exercise, supporting a direct role of EPO in the regulation of muscle energetics. Such a role is further supported by the observation that EPO alone is sufficient to increase PGC-1α expression, confirming similar results reported in healthy rats [41].

Regarding mitochondrial dynamics, our gene study shows that, while mitochondrial fission-related transcripts are not affected by exercise, combined or not with EPO, those related to mitophagy are increased, likely allowing the removal of damaged mitochondria. Such hypothesis is strengthened by the recent demonstration that PGC-1α plays a regulatory role on mitophagy in the skeletal muscle [21]. Moreover, our observations suggest that the exercise protocol adopted is well tolerated in LLC-bearing mice. Indeed when endurance exercise is excessive, mitochondrial fission increases in the absence of mitophagy induction [46]. Our results are partially confirmed by a study performed in APC Min/+ cachectic mice, where the reduction of mitochondrial content is associated with PGC-1α and mitochondrial fusion protein repression, that is effectively prevented by either IL-6 blockade or exercise [39]. Most of the studies describing muscle mitochondrial alterations confer to PGC-1α reduced expression a causative role. Consistently, PGC-1α overexpression has been shown to effectively prevent muscle atrophy in certain conditions [23], acting as an exercise mimetic. In agreement with these observations, the present study shows that PGC-1α overexpression proves effective in preventing LLC-induced muscle wasting. Such a result does not comply with a previous report suggesting that skeletal muscle hypertrophy and resistance to LLC-induced muscle wasting is exclusively regulated by PGC-1α4, a non-canonical resistance training-induced splice variant of the PGC-1α gene [47]. However, recent data obtained in humans question the specificity of PGC-1α4 in mediating exercise-induced hypertrophy, since all PGC-1α isoforms are induced after exercise regardless of the mode [48]. Nonetheless, the mode of exercise could be relevant to the effectiveness of such an approach in cancer patients, resistance favoring the maintenance of muscle mass and endurance improving muscle quality. It is likely that the integration of both components will provide the most beneficial effect.

**Figure 7:**
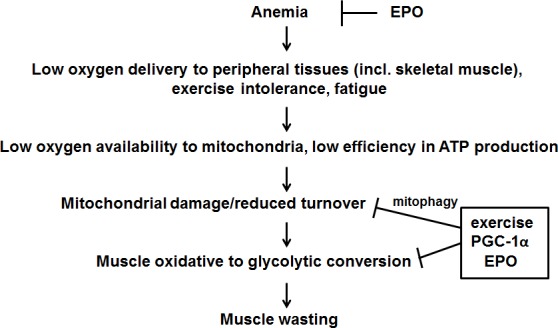
Proposed mechanism of action of exercise, EPO and PGC-1α in counteracting tumor-induced muscle alterations See text for further details.

In conclusion, our results highlight the importance of a combined approach in order to target at least some of the alterations occurring in cachectic patients (Figure 7). On a first line, EPO administration would prevent cancer-induced anemia and boost exercise effectiveness, counteracting fatigue. As for the skeletal muscle, the association of EPO administration to moderate exercise could promote, through stimulation of PGC-1α expression, both mitochondrial biogenesis and turnover, improving muscle quality and function. Such approach looks feasible and provides the scientific bases and mechanistic support for translating the preclinical data into clinical trials exploring, in a combined therapeutic strategy, the effectiveness of exercise for countering cachexia.

## MATERIALS AND METHODS

### Animals and experimental design

Male 6 week old Balb/C or C57BL/6 (Interfauna, Spain) and both male and female C57BL/6-Tg(Ckm-Ppargc1a)31Brsp/j (overexpressing PGC-1α1 in the skeletal muscle [49]; the Jackson Laboratory, USA) mice were maintained on a regular dark-light cycle (light from 08:00 to 20:00), with free access to food and water during the whole experimental period. Animals were cared for in compliance with the Policy on Human Care and Use of Laboratory Animals (ILAR 2011). The Bioethical Committees of the University of Barcelona and Torino approved the experimental protocols. All animal manipulations were made in accordance with the European Community guidelines for the use of laboratory animals.

Mice were randomized and divided into two groups, namely controls (C) and tumor bearers (TB). TB mice were inoculated subcutaneously in the back with 5×10^5^ Colon26 (C26) or Lewis Lung carcinoma (LLC) cells. C26 and LLC cells were maintained *in vitro* in DMEM (Invitrogen) supplemented with 10% FBS, 100 U/ml penicillin, 100 μg/ml streptomycin, 100 μg/ml sodium pyruvate, 2 mM L-glutamine, at 37°C in a humidified atmosphere of 5% CO_2_ in air. The day of tumor implantation, cells were trypsinized, resuspended in sterile saline, and implanted in the back of the animals. Tumor-bearing mice were divided into different groups: sedentary (C26 and LLC), submitted to endurance exercise (C26 EX and LLC EX), treated every three days with an intraperitoneal injection of recombinant human EPO (100 IU, see [50]; C26 EPO and LLC EPO) and submitted to endurance exercise and erythropoietin treatment (C26 EX EPO and LLC EX EPO). Transgenic mice were randomized and divided into two groups, namely controls (C PGC) and tumor bearers (LLC PGC). As for the exercise protocol, mice were exercised on a Panlab/Harvard-Apparatus treadmill (Barcelona, Spain). Mice were exercised 5 days/week starting the day after tumor injection for both C26 and LLC. In a subsequent experiment on C26 mice, the animals were exercised starting 6 weeks before tumor injection until sacrifice. Mice ran for 45 minutes at the speed of 14 m/minute (see [16] for details).

Animal weight and food intake were recorded daily, starting from the day of tumor implantation. Control and tumor-bearing mice were sacrificed under anesthesia 14 or 28 days after C26 or LLC implantation, respectively. Several muscles and tissues were rapidly excised, weighed, frozen in melting isopentane and stored at −80°C for further analysis.

### Grip force assessment

Muscle strength was assessed by the grip-strength test as previously described [16] using a Panlab-Harvard Apparatus device. Three measurements were taken for each mouse on both baseline and sacrifice day.

### Hematocrit

Blood was collected from anaesthetized mice by cardiac puncture. A drop was used to fill hematocrit capillary tubes that were centrifuged in a specific centrifuge for 5 min at 800 x g. Hematocrit was expressed as volume percentage of erythrocytes in the blood.

### Histology, SDH staining and total activity

During sacrifice, both tibialis muscles from each animal were rapidly excised. One was snap frozen in liquid nitrogen for enzymatic activity and western blotting, the other was mounted in OCT and frozen in melting isopentane for histology. Transverse sections (10 μM) were cut on a cryostat and stained for SDH (succinate dehydrogenase). Briefly, cryosection were incubated for 30 min at 37°C with 1 mg/ml NTB (nitrotetrazolium blue chloride) and 27 mg/ml Na-succinate in PBS, washed three times in PBS, mounted with glycerol and photographed. Fiber cross-sectional area (CSA) was determined using the Image J software on randomly chosen 100 individual fibers in the two regions of the muscle composed mainly by oxidative or glycolytic fibers, respectively. As for the total SDH activity, the muscles were homogenized (5% wt/vol) in ice-cold 150 mM NaCl, 10 mM KH2PO4, 0.1 mM EGTA and centrifuged 5 min at 800 x g. The supernatant was collected and total protein content measured using the BCA protein assay (Pierce, Thermo Fisher Scientific, USA). Protein homogenates (50 μl) were incubated with 200 μl reaction buffer containing 10 mM Na-succinate, 50 μg/ml DCPIP, 10 mM phosphate buffer (pH 7.4), 2 mM KCN, 10 mM CaCl2, 0.05% BSA. The absorbance at 600 nm was measured after 0, 3 and 20 min. The rate of absorbance decrease between 3 and 20 min was corrected for the protein loading and used to calculate the SDH content.

### Electron microscopy

EDL and soleus muscle specimens of 1 mm^3^ were prepared under a stereomicroscope and fixed for 24 h at 4°C with 2% parafomaldehyde and 2.5% glutaraldehyde in phosphate buffer. After washing, samples were postfixed with 1% osmium tetroxide and 0.8% potassium ferricyanide at 4°C and finally dehydrated in acetone, infiltrated with Epon resin during 48 h, embedded in the same resin positioned to obtain longitudinal sections and polymerised at 60°C during 48 h. After semi-thin sectioning and field selection, ultrathin sections were obtained using a Leica Ultracut UC6 ultramicrotome and mounted on Formvar-coated copper grids. Sections were stained with 2% uranyl acetate in water and lead citrate and observed in a JEM-1010 electron microscope (Jeol, Japan) equipped with a CCD camera SIS Megaview III and the AnalySIS software. Intermyofibrillar mitochondrial morphology was classified into unchanged and altered (swelling-related ultrastructural changes). Mitochondrial counting was performed computing the average number of altered mitochondria in 3 randomly taken micrographs per sample and 3 samples per experimental group.

### Intracellular ATP content

ATP was determined by bioluminescence using a commercially available kit (ATP Bioluminescence Assay Kit CLS II; Roche) according to manufacturer's recommendations. Briefly, gastrocnemius muscles were homogenized in PBS (10% wt/vol). Muscle homogenates were then diluted 10 times in 100 mM Tris, 4 mM EDTA (pH 7.75), incubated 2 minutes at 100°C, and centrifuged 1 minute at 1000 x g and the supernatant collected. An aliquot of sample (50 μl) was added to 50 μL of the luciferase reagent in a multiwell black plate (96 wells - Packard). The luminescence was measured in a Luminometer at 562 nm with an integration time of 10 seconds. ATP concentrations were obtained from a log-log plot of the standard curve data.

### Western blotting

Tibialis muscles were homogenized in 10 mM HEPES, pH 7.5, containing 10 mM MgCl2, 5 mM KCl, 0.1mM EDTA and 0.1% Triton X-100, with freshly added protease and phosphatase inhibitor cocktails, centrifuged at 3000 x g for 5 min at 4°C, and the supernatant collected as cytosolic extract. The pellet was resuspended in 20 mM HEPES, pH 7.9, containing 1.5 mM MgCl2, 500 mM NaCl, 0.2mM EDTA and 25% glycerol, with freshly added protease and phosphatase inhibitor cocktails, incubated on ice for 30 min, centrifuged at 3000 x g for 5 min at 4°C, and the supernatant collected as nuclear extract. Protein concentration was measured using the BCA protein assay (Pierce, Thermo Fisher Scientific, USA). Equal amounts of nuclear protein (20 μg) were heat-denaturated in sample-loading buffer (50 mM Tris-HCl, pH 6.8, 100 mM DTT, 2% SDS, 0.1% bromophenol blue, 10% glycerol), resolved by SDS-PAGE and transferred to nitrocellulose membranes (Bio-Rad, USA). The filters were blocked with Tris-buffered saline (TBS) containing 0.05% Tween and 5% non-fat dry milk and then incubated overnight with an antibody directed against PGC-1α (Millipore, USA). Peroxidase-conjugated IgG (Bio-Rad, USA) were used as secondary antibodies. Membrane-bound immune complexes were detected by an enhanced chemiluminescence system (Santa Cruz Biotechnology, USA) on a photon-sensitive film (Hyperfilm ECL, GE Healthcare, Italy). Protein loading was normalized according to GAPDH (Santa Cruz biotechnology, USA) expression. Quantification of the bands was performed by densitometric analysis using a specific software (TotalLab, NonLinear Dynamics, UK).

### RT-PCR

Total RNA was obtained using the TriPure reagent (Roche, USA) following manufacturer's instructions. RNA concentration was determined fluorometrically using the Ribogreen reagent (Invitrogen, USA). Total mRNA was retro-transcribed using the i-Script cDNA synthesis kit (Bio-Rad, USA). Transcript levels were determined by real-time PCR using the SsoFast Evagreen Supermix and the MiniOpticon thermal cycler (Bio-Rad, USA). Primer sequences are given in the supplemental material section.

### Data analysis and presentation

All the results are expressed as means ± SD, except for gene expression (means ± SEM). The significance of the differences was evaluated by analysis of variance (ANOVA) followed by Tukey's test.

## SUPPLEMENTARY MATERIALS AND METHODS FIGURES AND TABLE


