# Supplementary material for: Combination of exercise training and erythropoietin prevents cancer-induced muscle alterations
Source: Oncotarget. 2015 Nov 30;6(41):43202–15. doi: 10.18632/oncotarget.6439 (PMC4791226; doi:10.18632/oncotarget.6439)
Supplement: Supplementary file 1 [file oncotarget-06-43202-s001.pdf]

Combination of exercise training and erythropoietin prevents cancer-induced muscle alterations

Supplementary material and methods:

IL-6 ELISA:

IL-6 serum levels were detected by a commercially available mouse ELISA kit, used according to the manufacturer instructions (Bender MedSystems, Austria). Serum from each animal (50 µl) was assayed in duplicate. Quantitative calibration was obtained performing a standard curve with recombinant mouse IL-6.

- Primer sequences

EPOR
AAGTCAGACGGGCAGGAAGAT AGTGAGCATGCCCAGGACA
NM_010149.3
Mb
AGTGATGGGGAGTGGCAGCTG ACCATGCTTCTTCAGGTCCTCTGA
NM_001164048.1
Atrogin-1
GCTTCCCCCAAAGTGCAGTA CCATCAGGAGAAGTGGATCTATGTT
NM_026346.3
PGC1α
GAGTCTGAAAGGGCCAAACA TGCATTCCTCAATTTCACCA
NM_008904.2
PGC1β
GGACGCCAGTGACTTTGACT TTCATCCAGTTCTGGGAAGG
NM_133249.2
NRF-1
GAACTGCCAACCACAGTCAC TTTGTTCCACCTCTCCATCA
NM_010938.4
NIX (BNIP3L)
TTGGGGCATTTTACTAACCTTG TGCAGGTGACTGGTGGTACTAA
NM_009761.3
BNIP3
TTCCACTAGCACCTTCTGATGA GAACACCGCATTTACAGAACAA
NM_009760.4
DRP1
GTTCCACGCCAACAGAATAC CCTAACCCCCTGAATGAAGT
NM_001025947.1
Fis1
AAGTATGTGCGAGGGCTGT TGCCTACCAGTCCATCTTTC
NM_001163243.1
MuRF-1

TCCTGCCGAGTGACCAAGGAAA	AGGATGGCGTAGAGGGCGTCAA
NM_001039048.2	
**TBP**	
CCCCACAACTCTTCCATTCT	GCAGGAGTGATAGGGGTCAT
NM_013684.3	
**β-Actin**	
GACATGGAGAAGATCTGGCA	GGTCTCAAACATGATCTGGGT
NM_007393.3	

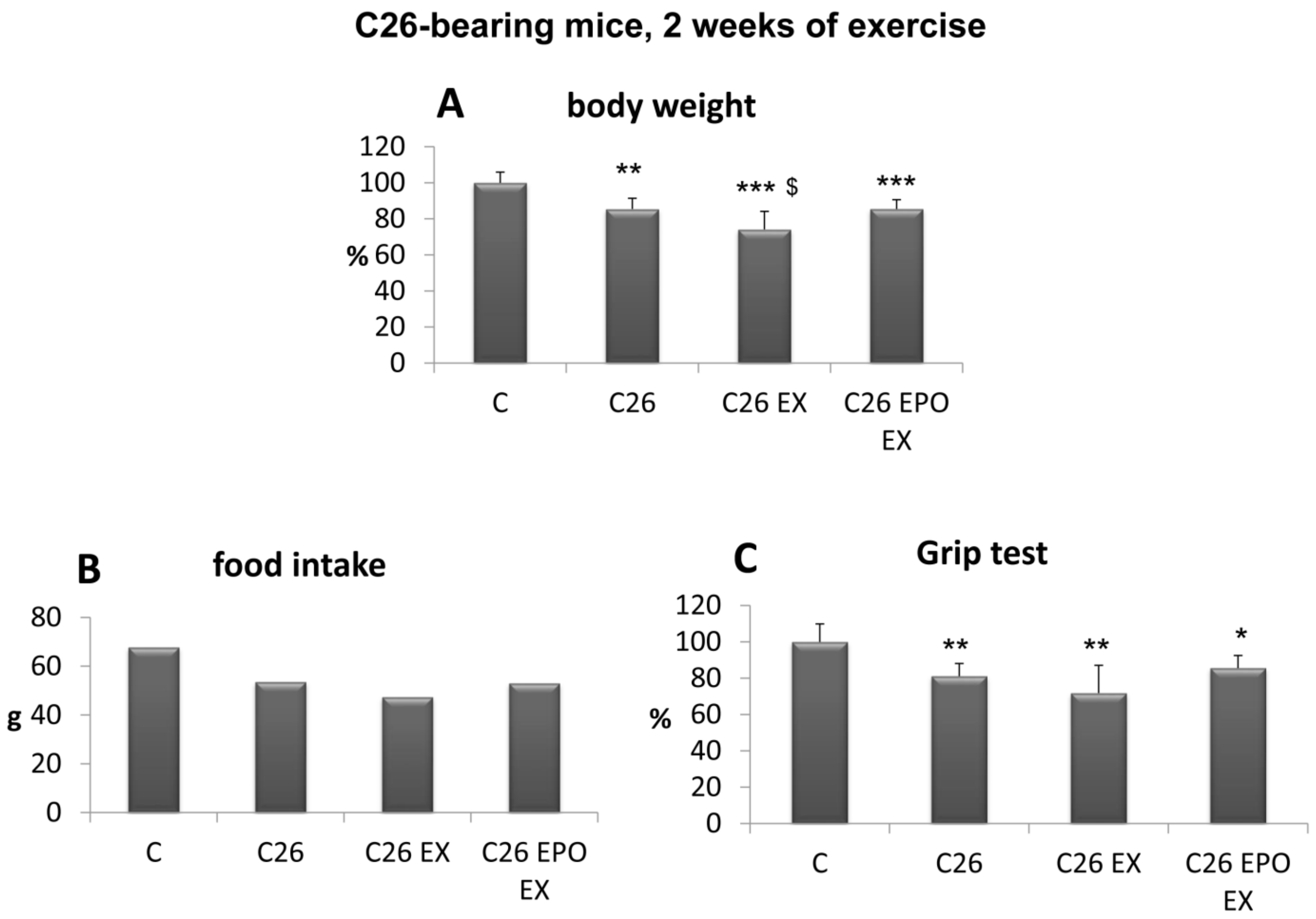

Fig. S1

Body weight (**A**), voluntary grasping strength (**B**) and cumulative food intake (**C**), in control (C), and C26-bearing mice (C26). C26 groups were subdivided in untreated, exercised (EX), and exercised EPO-treated (EX EPO) for two weeks. Significance of the differences: *p < 0,05 vs C, **p < 0,01 vs C, ***p < 0,001 vs C, $p < 0,05 vs C26.

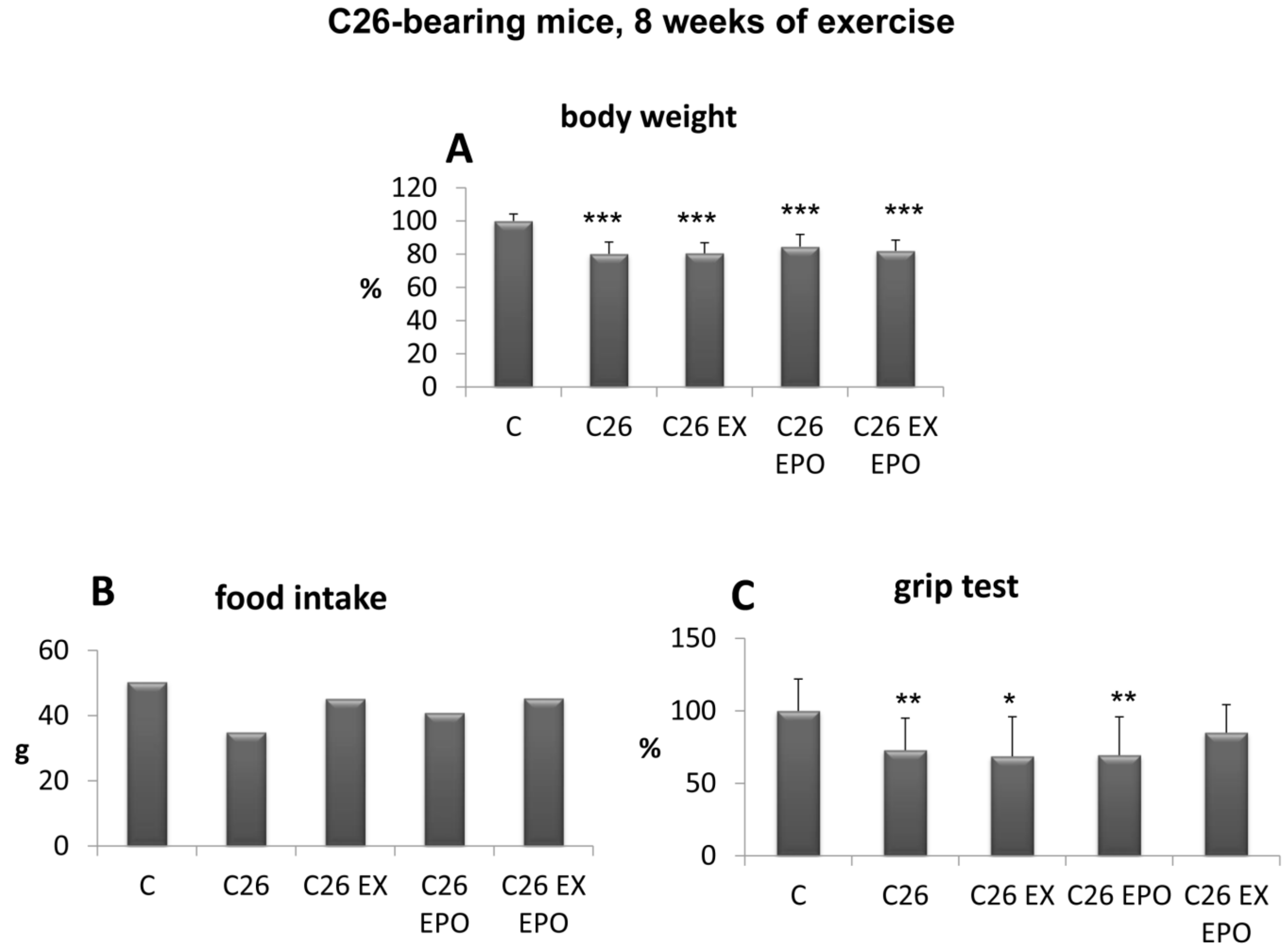

Fig. S2

Body weight (**A**), voluntary grasping strength (**B**) and cumulative food intake (**C**), in control (C), and C26-bearing mice (C26). C26 groups were subdivided in untreated, exercised (EX), EPO-treated (EPO) and exercised EPO-treated (EX EPO) for eight weeks (of exercise). Significance of the differences: *p < 0,05 vs C, **p < 0,01 vs C, ***p < 0,001 vs C.

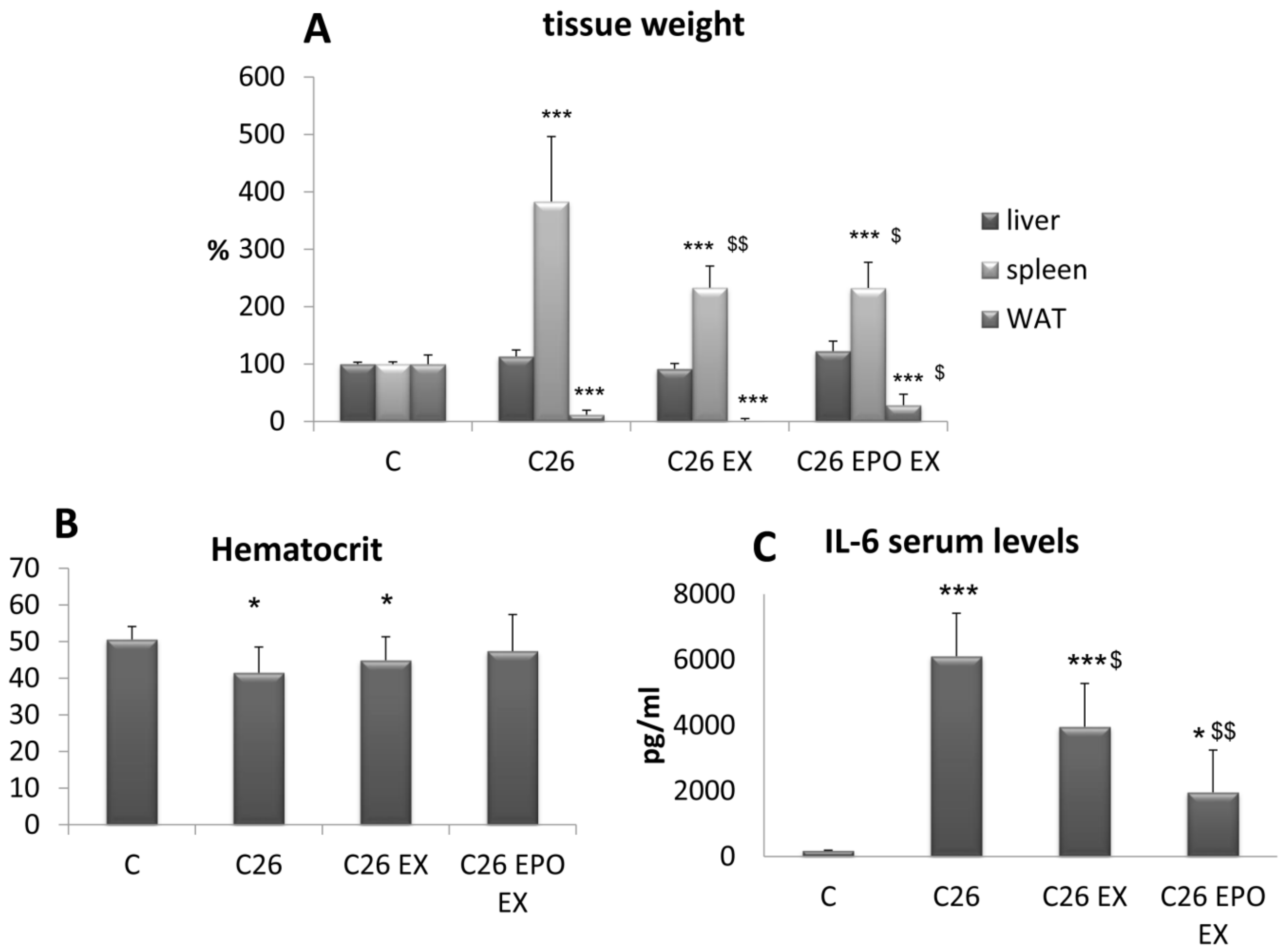

Fig. S3

Liver, spleen and white adipose tissue (WAT) weight (**A**), hematocrit (**B**) and circulating IL-6 levels (**C**) in control (C) and C26-bearing mice (C26). C26 groups were subdivided in untreated, exercised (EX), and exercised EPO-treated (EX EPO) for two weeks. Data (mean±SD) expressed as percentages of C except for hematocrit (absolute value). Significance of the differences: *p < 0,05 vs C, ***p < 0,001 vs C, $p < 0,05 vs C26, $$p < 0,01 vs C26.

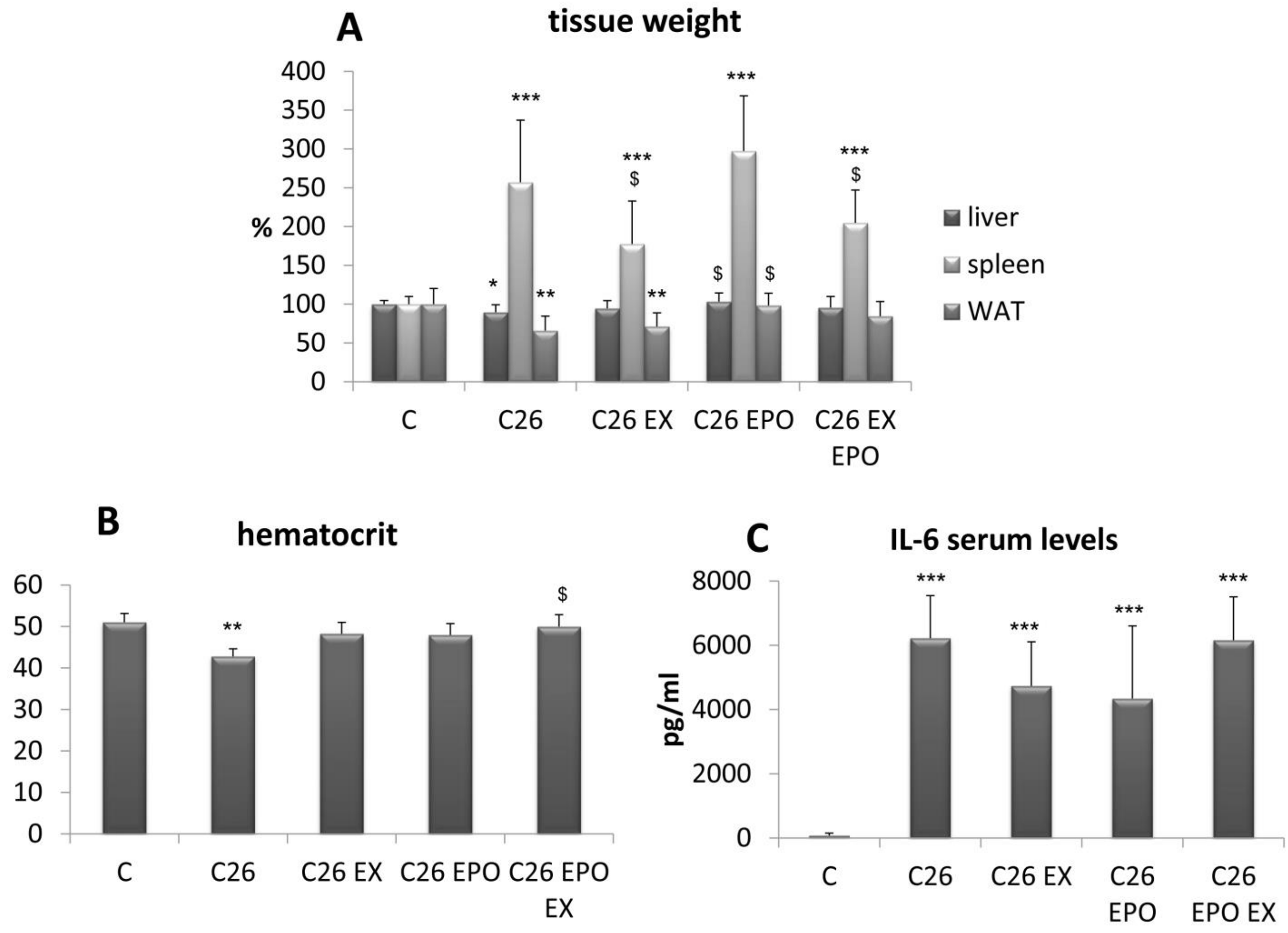

Fig. S4

Liver, spleen and white adipose tissue (WAT) weight (**A**), hematocrit (**B**) and circulating IL-6 levels (**C**) in control (C) and C26-bearing mice (C26). C26 groups were subdivided in untreated, exercised (EX), and exercised EPO-treated (EX EPO) for eight weeks (of exercise). Data (mean±SD) expressed as percentages of C except for hematocrit (absolute value). Significance of the differences: *p < 0,05 vs C, **p < 0,01 vs C, ***p < 0,001 vs C , $p < 0,05 vs C26.

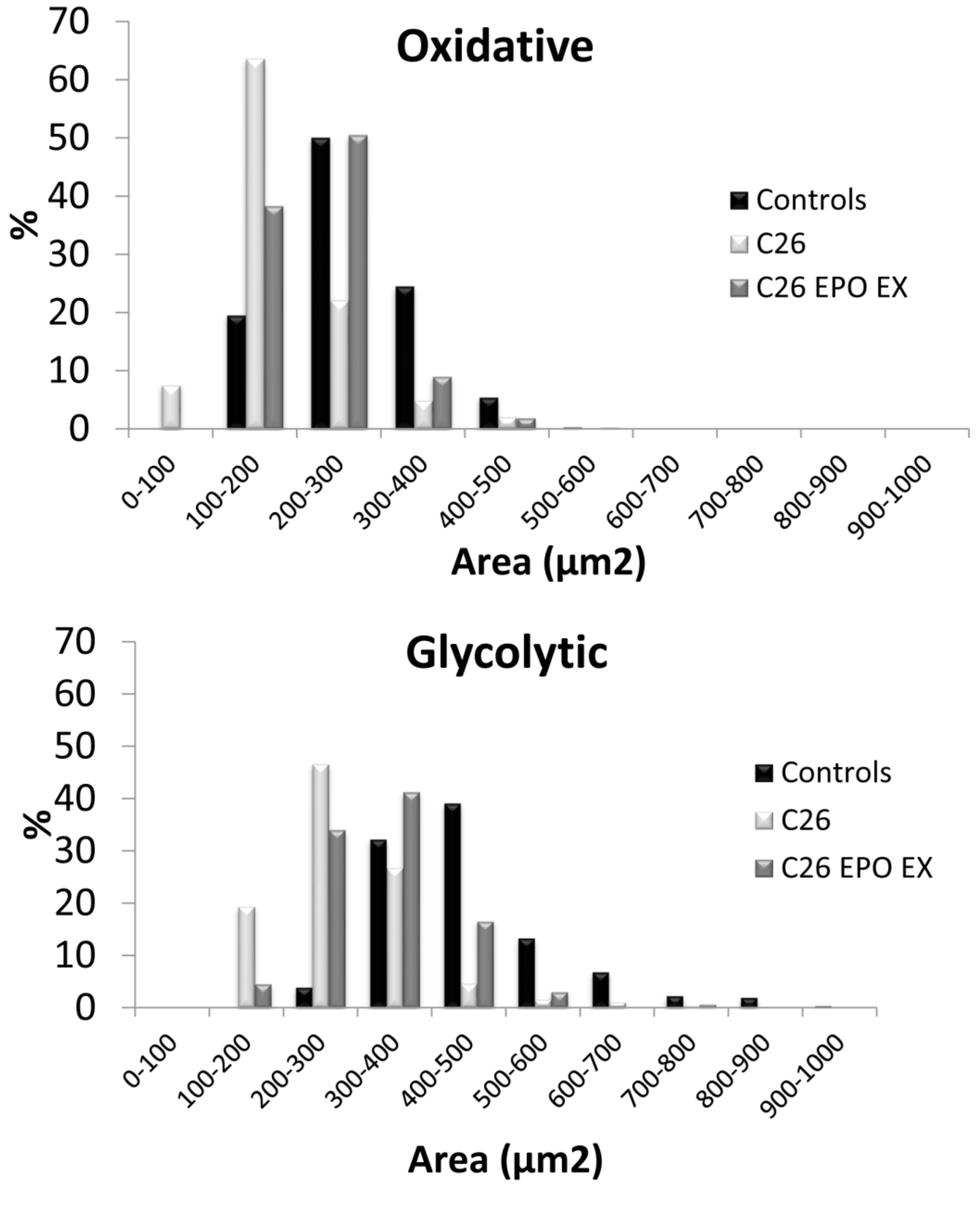

Fig. S5

Morphometric analysis of myofiber CSA (cross-sectional area) performed on SDH stained sections of tibialis muscle from control (C), C26-bearing (C26) and C26 exercised EPO-treated (EX EPO) mice (2 weeks of exercise). CSA were independently measured in oxidative and glycolytic fibers. Data are expressed as percentage of frequency distribution. Average fibers measured per muscle: C= 224; C26= 279; C26 EX EPO= 232.

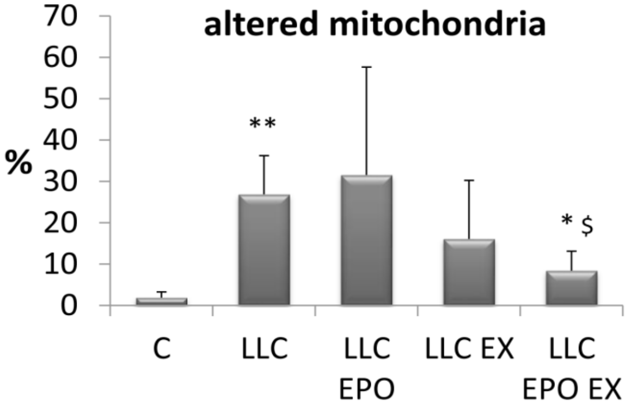

Fig. S6

Quantification of altered mitochondria in EDL (*Extensor digitorum longus*) sections, expressed as % of altered mitochondria/total mitochondria in control (C) and LLC-bearing mice (LLC). LLC groups were subdivided in sedentary, EPO-treated (EPO), exercised (EX), and exercised EPO-treated (EX EPO). Significance of the differences: *p < 0,05 vs C, **p < 0,01 vs C, $p < 0,05 vs LLC.

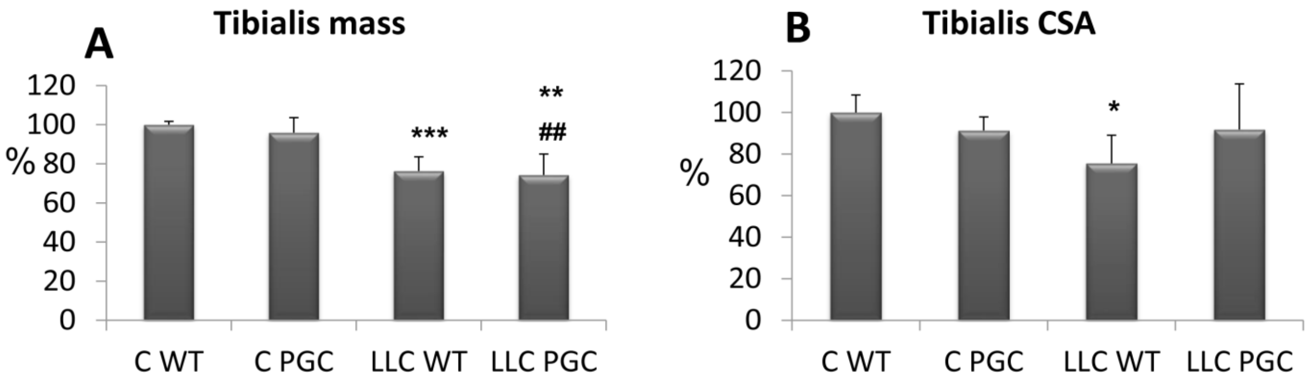

Fig. S7

Tibialis weight (**A**) and fiber CSA (**B**) in male control WT (C WT), PGC (C PGC) and LLC-bearing mice WT (LLC WT) and PGC (LLC PGC). Data (mean±SD) expressed as percentages of C WT. Data (mean±SE) are compared by 2-way ANOVA. Significance of the differences: *p < 0,05 vs C WT, **p < 0,01 vs C WT, ***p < 0,001 vs C WT, ##p < 0,01 vs C PGC.

Supplementary Table1

n size of mice in the different experimental groups at the beginning and at the end of the 4 week experimental period of LLC tumor growth. Tumor mass is expressed as mean ± SD. No statistically significant difference was found among the groups for both survival and tumor mass.

**Experimental group**	n (starting point)	n (end point)	Tumor mass (g)
**C**	8	8	
**LLC**	8	7	5.59 ± 0.33
**LLC EPO**	8	6	6.44 ± 1.48
**LLC EX**	8	7	4.62 ± 0.37
**LLC EPO EX**	8	8	5.22 ± 0.46
